# Usefulness of a New Three-Dimensional Simulator System for Radiofrequency Ablation

**DOI:** 10.1371/journal.pone.0148298

**Published:** 2016-02-04

**Authors:** Masashi Hirooka, Yohei Koizumi, Yusuke Imai, Teruki Miyake, Takao Watanabe, Osamu Yoshida, Eiji Takeshita, Yoshio Tokumoto, Masanori Abe, Yoichi Hiasa

**Affiliations:** Department of Gastroenterology and Metabology, Ehime University Graduate School of Medicine, Shitsukawa, Toon, Ehime, Japan; Taipei Veterans General Hospital, TAIWAN

## Abstract

Multipuncture radiofrequency ablation is expected to produce a large ablated area and reduce intrahepatic recurrence of hepatocellular carcinoma; however, it requires considerable skill. This study evaluated the utility of a new simulator system for multipuncture radiofrequency ablation. To understand positioning of multipuncture electrodes on three-dimensional images, we developed a new technology by expanding real-time virtual ultrasonography. We performed 21 experimental punctures in phantoms. Electrode insertion directions and positions were confirmed on computed tomography, and accuracy and utility of the simulator system were evaluated by measuring angles and intersections for each electrode. Moreover, to appropriately assess placement of the three electrodes, puncture procedures with or without the simulator were performed by experts and non-experts. Technical success was defined as maximum angle and distance ratio, as calculated by maximum and minimum distances between electrodes. In punctures using 2 electrodes, correlations between angles on each imaging modality were strong (ultrasound vs. simulator: r = 0.991, p<0.001, simulator vs. computed tomography: r = 0.991, p<0.001, ultrasound vs. computed tomography: r = 0.999, p<0.001). Correlations between distances in each imaging modality were also strong (ultrasound vs. simulator: r = 0.993, p<0.001; simulator vs. computed tomography: r = 0.994, p<0.001; ultrasound vs. computed tomography: r = 0.994, p<0.001). In cases with 3 electrodes, distances between each electrode correlated strongly (yellow-labeled vs. red-labeled: r = 0.980, p<0.001; red-labeled vs. blue-labeled: r = 0.953, p<0.001; yellow-labeled vs. blue-labeled: r = 0.953, p<0.001). Both angle and distance ratio (expert with simulator vs. without simulator; p = 0.03, p = 0.02) were significantly smaller in procedures performed by experts using the simulator system. The new simulator system appears to accurately guide electrode positioning. This simulator system could allow multipuncture radiofrequency ablation to be performed more effectively and comfortably.

## Introduction

**Fig 1:**
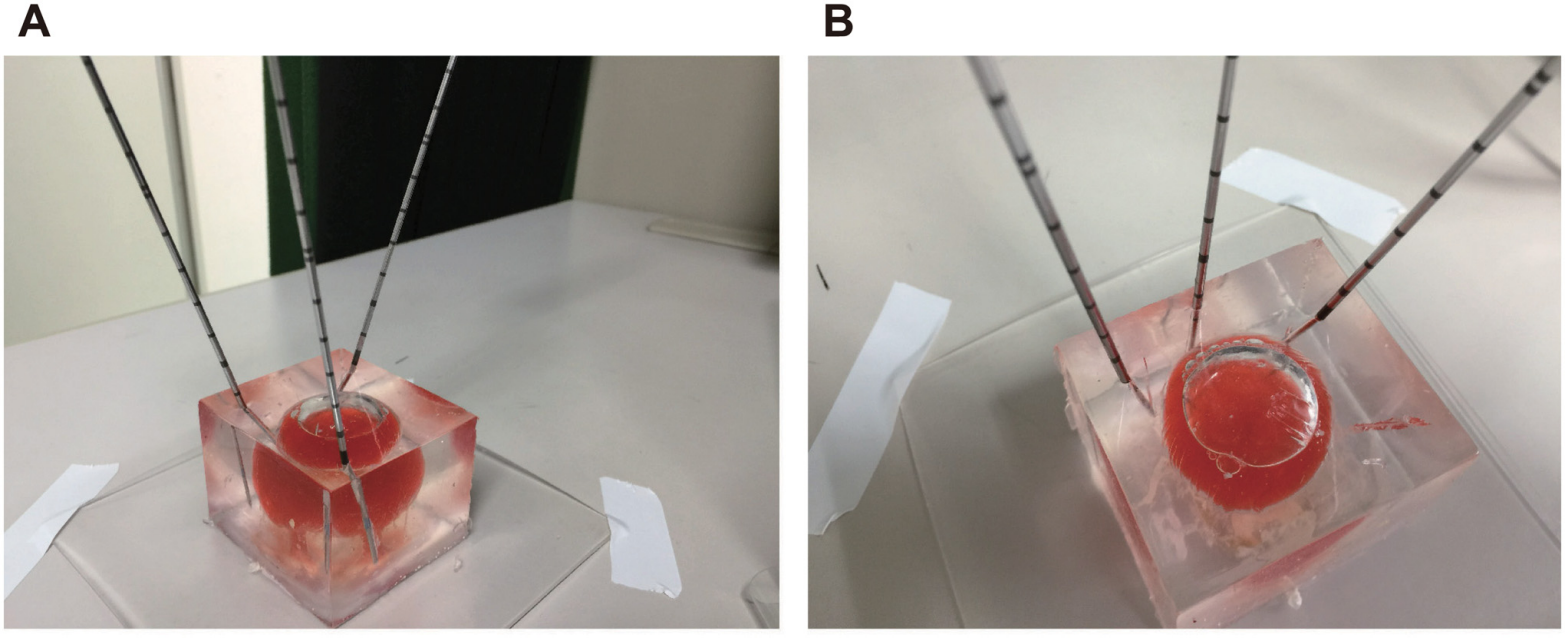
Multipuncture procedure in a gelatin model. Parallel placement of the three electrodes at regular intervals (**A**), resulted in all electrodes being positioned inappropriately (**B**). If the multipuncture procedure is performed using only 2-dimensional ultrasound, electrodes often display positioning biased toward one side.

**Fig 2:**
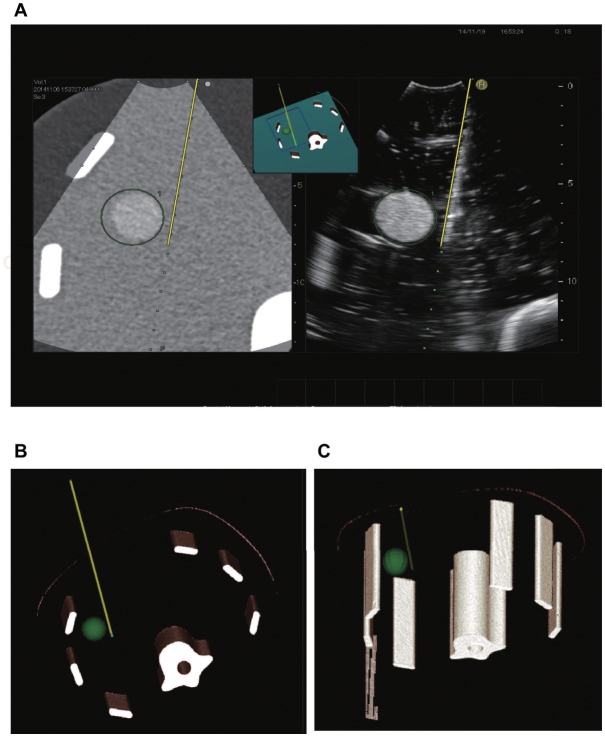
Virtual puncture line constructed using the new simulator system. **A)** Display of real-time virtual sonography. After puncture, the RF electrode is marked with a yellow line in the right image. This line is reflected in the left multiplanar reconstruction (MPR) image as the same slice section in the right ultrasound image. **B, C)** This line can be depicted on 3D images and can be observed from various angles.

In radiofrequency ablation (RFA), an ablated margin of >5 mm is needed to achieve therapeutic results similar to those achieved with hepatectomy [1–4]. Monopolar RF ablation technology shows limited ability to reliably create an adequate volume of coagulation necrosis [1]. To achieve an adequate safety margin using a monopolar electrode, multiple overlapping ablations are required over several sessions [5]. Repositioning the monopolar electrode under ultrasonographic guidance is very difficult because the changes in the hyperechoic area induced by the previous ablation may interfere with positioning the tip of the electrode and identifying unablated residual tumor [6]. Recently, a multipolar ablation system with up to three bipolar electrodes has become available [7–10]. Multipuncture ablation can produce a large ablated area, but appropriate insertion of multiple electrodes around the tumor is very difficult, even for experts [11]. Since percutaneous procedures are often performed under two-dimensional (2D) ultrasonography, well-balanced placement of electrodes around the tumor is sometimes not achieved (Fig 1). Particularly in tumors smaller than 3 cm in diameter, multipolar ablation without direct puncture (so-called “no-touch ablation”) is often performed. No-touch ablation may diminish the risk of tumor seeding [12–14] and intraperitoneal hemorrhage, since the electrodes are not directly inserted into the hepatocellular carcinoma (HCC) nodule [11]. However, since the insertion of electrodes is performed without direct puncture, recognizing well-balanced placement of electrodes around the tumor is quite challenging. Navigation systems should thus be used to facilitate appropriate insertion of the electrodes. A previous study reported the efficacy of using a virtual puncture line on three-dimensional (3D) images for patients with HCC of the caudate lobe [15]. A virtual puncture line is useful for simulating the puncture route of a single electrode in a 3D manner. To clearly understand positioning of the electrodes in the 3D space, we developed a simulator system to improve on the concept of the virtual puncture line (Fig 2).

This study evaluated the efficacy of the simulator system by analyzing its accuracy in a phantom study.

## Materials and Methods

### Simulator system evaluation in a phantom

**Fig 3:**
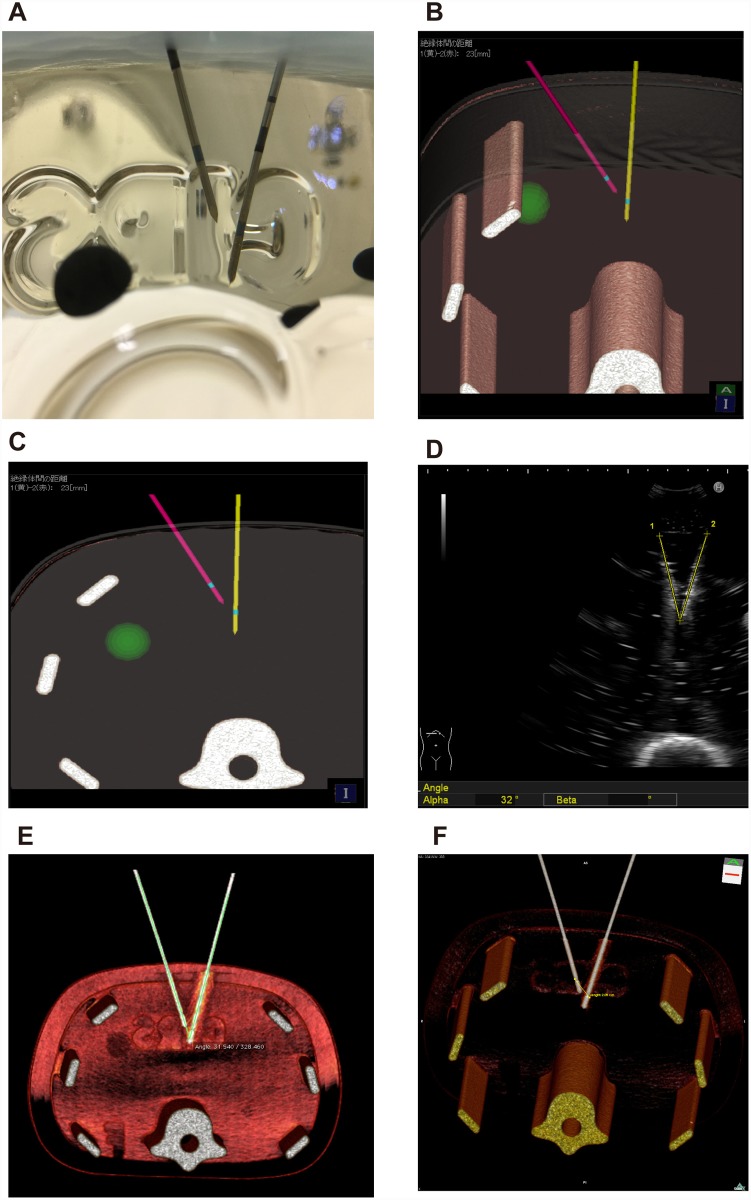
Analysis with insertion of 2 electrodes. **A)** External appearance of the phantom after puncture. **B)** Distance between the 2 electrodes (white arrow). **C)** Distance between the 2 electrodes as measured on CT. **D-F)** The angle of intersection of the 2 electrodes as measured on the simulator system (**D**), ultrasonography (**E**), and CT (**F**).

**Fig 4:**
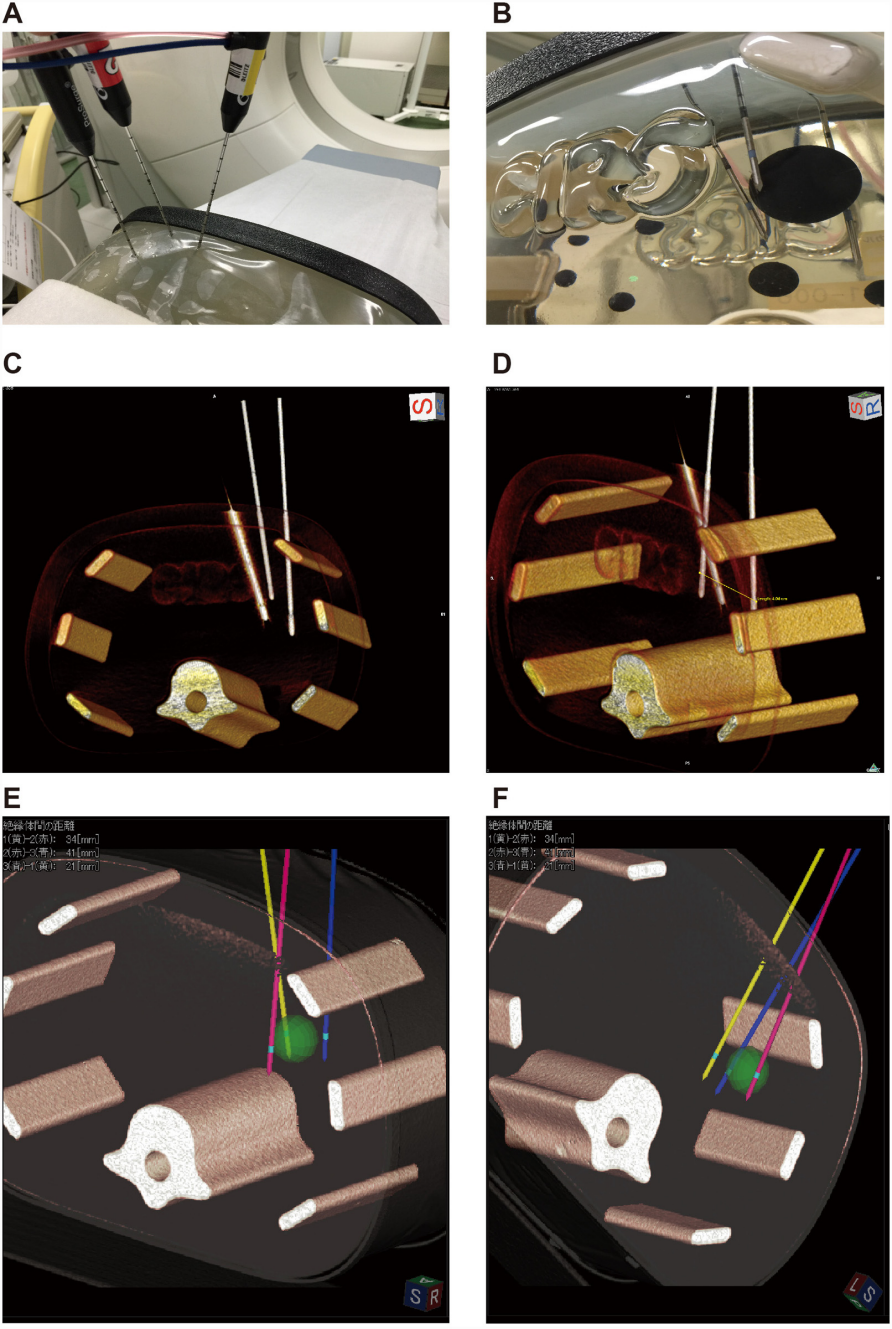
Analysis after puncture with 3 electrodes. **A, B)** External appearance in the phantom study. **C)** Appearance on 3D-CT. This image largely corresponds to **B**. **D, E)** Distances measured on CT (**D**) and the simulator system (**E**) between the 3 electrodes.

**Fig 5:**
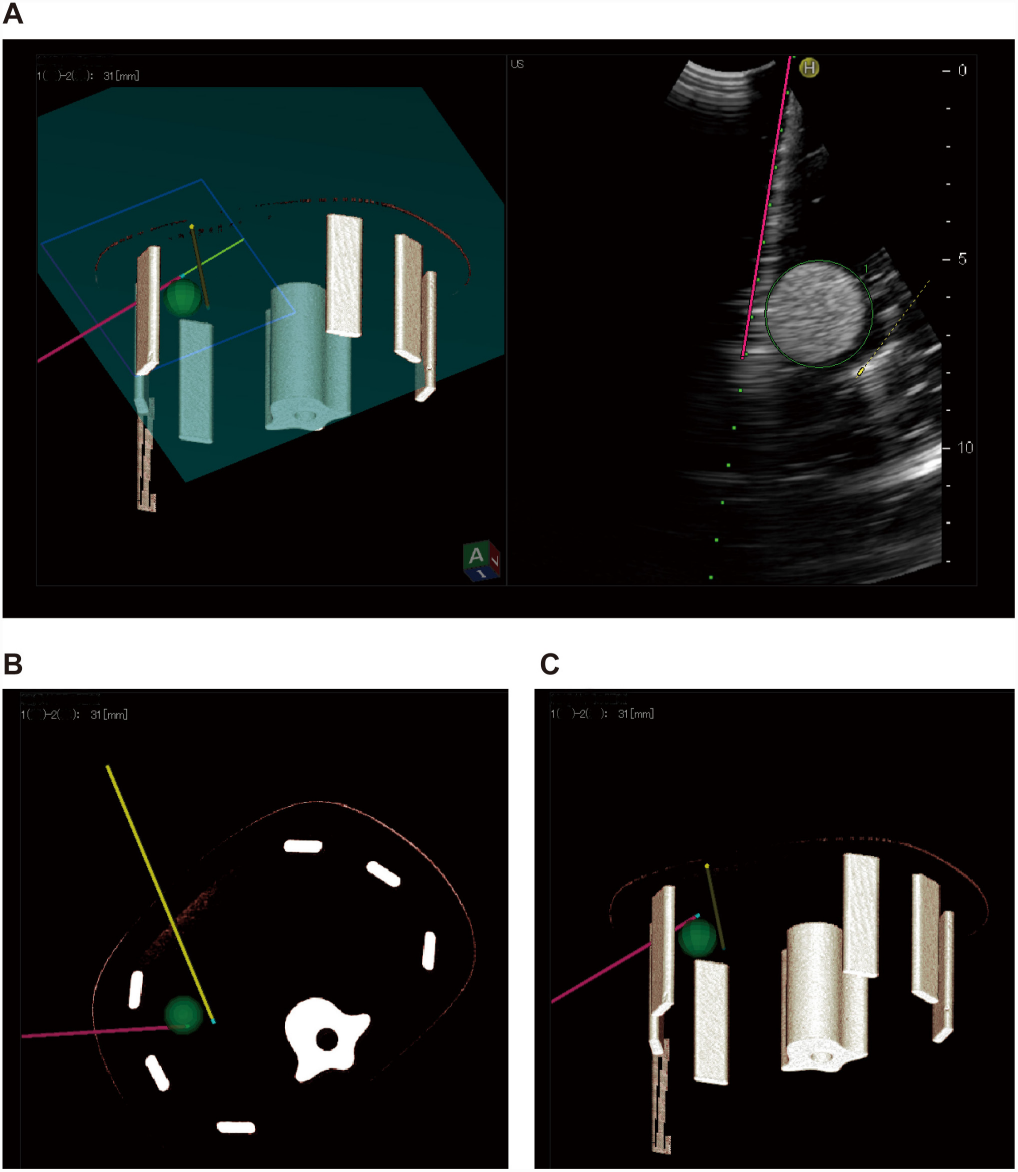
Insertion of the second RF electrode. **A)** The first electrode is inserted from the left ventral side of the phantom (yellow line), represented as the green ball in the left image. The blue plane represents the direction of ultrasonic scanning. The B-mode image is depicted on the right. The green dotted line in the B mode image corresponds to the light green line on the 3D image. The second RF electrode is shown as a pink line in the 3D image (A: left image) and by the marking electrode in the right B-mode image after puncture (A: right image). **B, C)** Both first and second RF electrodes are represented in the 3D image of the phantom.

**Fig 6:**
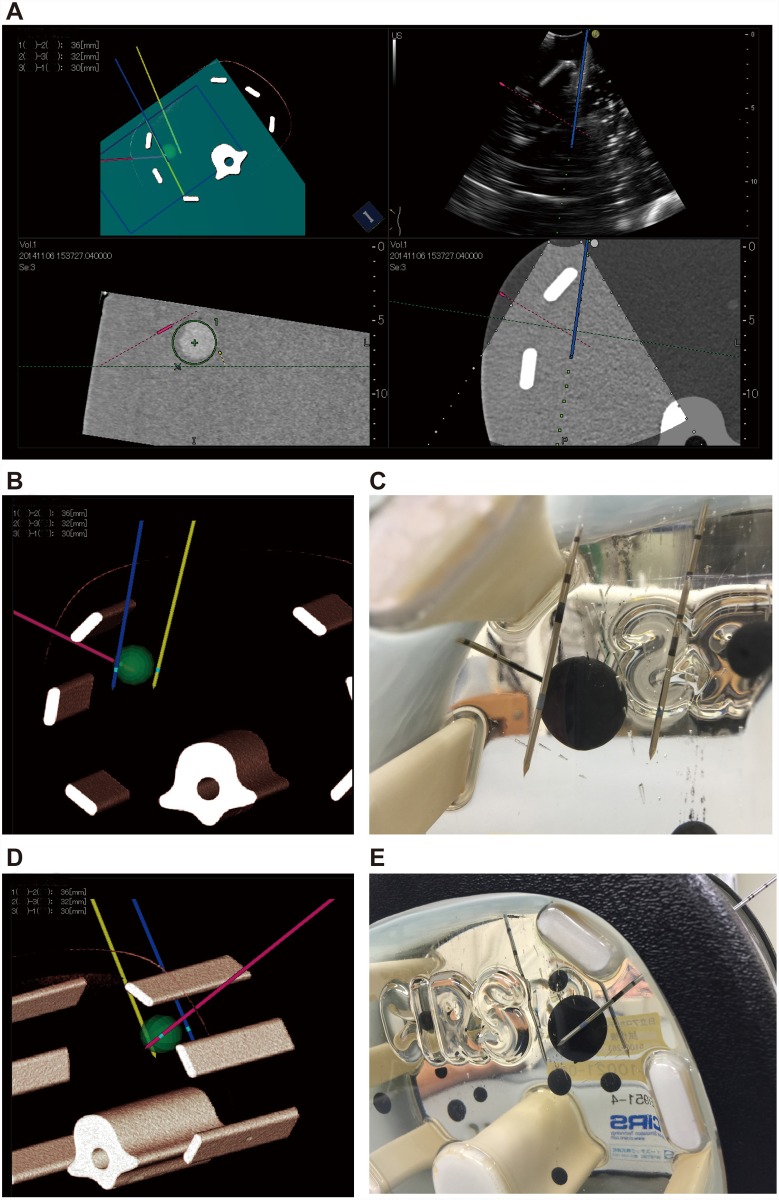
Representation of the three electrodes and the C-plane. **A)** The first electrode (yellow line) is shown on the left dorsal side of the tumor. The second electrode (pink line) can be recognized on the ventral side. To insert a third electrode on the right dorsal side of the tumor, the blue plane is matched to the appropriate direction. The C-plane is demonstrated in the lower left image. This image is defined as orthogonal to the blue plane of the upper left image. The green X represents the intersection between the light green line and the vertical plane. The direction of insertion of the third electrode is easily understood from this image. A B-mode image (upper right) and virtual sonographic image (lower left) are shown. **B, C)** The three electrodes seen from the caudal side are represented on the simulator image (**B**) and the phantom (**C**). **D, E)** These electrodes are observed from the cranial side on the simulator image (**D**) and the phantom (**E**).

**Fig 7:**
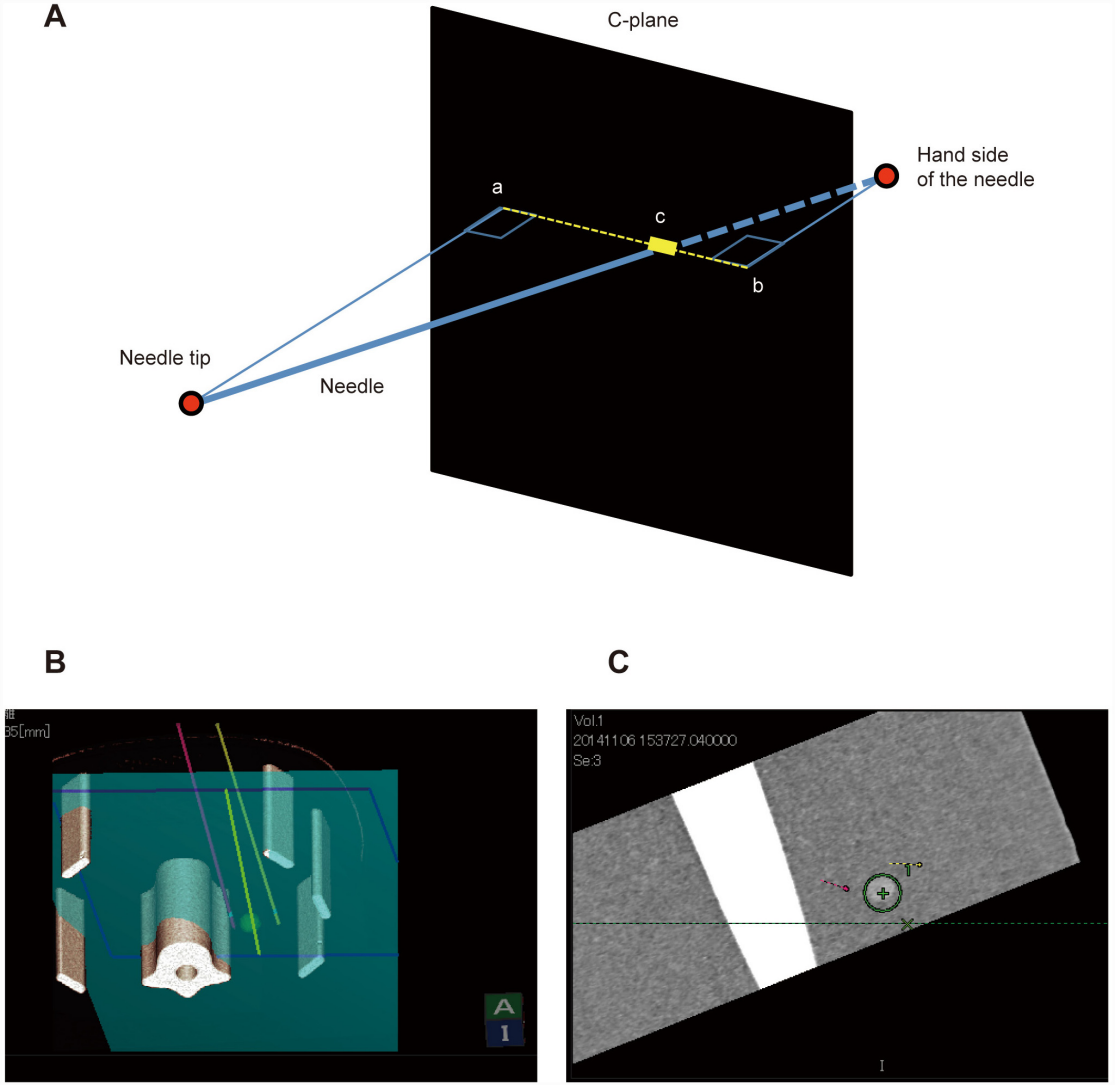
Principle of the C-plane. **A)** This schema shows the principle of the coronal plane (C-plane). The C-plane is a sagittal plane in relation to the original 2D multiplanar reconstruction image. The puncture line intersecting the C-plane is presented as a solid line (**C**). Sites a and b are projected on the C-plane from both ends of the puncture line. The dotted line is a straight line joining sites a and b. **B)** The first electrode (yellow line) is shown on the left dorsal side of the tumor, and the second electrode (pink line) can be recognized on the right dorsal side. To insert the third electrode on the ventral side of the tumor, the blue plane is matched to the appropriate direction. **C)** Demonstration of the C-plane. This image is defined as a sagittal view of the blue plane on the simulator image. The green X represents the intersection between the light green line and the sagittal plane.

To assess the simulator system’s utility, it was used to perform punctures in an abdominal biopsy phantom (CIRS 071; Supertech, Elkhart, IN). Digital imaging and Communications in Medicine (DICOM) data (as standard for distributing and viewing any kind of medical image regardless of origin) for the phantom were acquired from computed tomography (CT) performed using a 16-detector row scanner (Light Speed Ultra 16; GE Healthcare, Tokyo, Japan). Scan parameters were as follows: detector collimation, 16×0.63 mm; reconstruction thickness, 5-mm; scan pitch, 0.6°; and gantry rotation speed, 0.5 seconds [16]. Images for real-time virtual ultrasonography (RVS) were constructed using the DICOM data. An ultrasound device (Hi-Vision Preirus; Hitachi Aloka Medical, Tokyo, Japan) with RVS capability was used. The RVS system consists of a transmitter and magnetic sensor to detect the position of the sonographic probe. The virtual puncture line was constructed using a simulator system (3D-sim-Navigator; Hitachi Aloka Medical, Tokyo, Japan), which was developed by improving on the RVS system. First, the same cross-sectional multiplanar reconstruction (MPR) images as ultrasound images on the same monitor screen were obtained in real time, using DICOM volume data from CT (Fig 2A). Registration of the ultrasound (US) images with the cross-sectional MPR images was performed, starting from a reference plane that included the target tumor (usually axial plane). Moreover, to acquire good 2D correlations between US and MPR images, an active tracker (omniTRAX; CIVCO Medical Solutions, South Kalona, IA) was also used. The active tracker provides the operators with automatic image registration for fused images. To ensure valid registration, we confirmed whether the center of the tumor depicted on US corresponded exactly to that on MPR imaging. Several concordant landmarks, such as other tumor models in the phantom, were used to adjust the two displayed planes so that they overlapped completely. Next, the RFA electrode (Celon ProSurg; Celon AG Medical Instruments, Teltow, Germany) was marked on both MPR and B-mode images after insertion of the electrode into the liver of the phantom (Fig 2A). The marked line was defined as the virtual puncture line. Finally, the virtual puncture line was depicted on a 3D-CT image (Fig 2B and 2C). For testing on the phantom, 2 bipolar electrodes were inserted into the phantom (Fig 3). Punctures with the 2 electrodes were repeated 11 times at different sites. Punctures with three electrodes were also performed and repeated 10 times at different sites (Fig 4). The method for insertion of the second and third electrodes is shown in Figs 5 and 6. All the electrodes were clearly visible from various angles on the 3D images. CT was performed immediately after puncture with the 2 electrodes in the phantom, and the angle and distance between the two electrodes was measured (Fig 3). Measurement of the distance between the electrodes was performed at the level of the insulators on the electrodes, and their mean values were used for analysis. These measurements were performed on all three imaging studies, ultrasonography, simulator system and CT-MPR. For both ultrasonography and the simulator system, measurements were performed by M.H. and Y.K. Y.I. and T.M obtained the data on mean angles and distances on MPR-CT images (Fig 3). The two readers were blinded to each other's findings. Similarly, after puncture by the 3 electrodes, distances were measured between each electrode on the images from the simulator system and MPR (Fig 4). Moreover, to achieve insertion of the 3 electrodes in a triangular configuration, we developed a display named the C-plane (Fig 7), a sagittal plane in relation to the original 2D-MPR image synchronized to the B-mode image by RVS. The concept of the C-plane facilitates understanding of the positions at which all electrodes penetrate the tumor at the maximum cross-sectional area of the tumor (Fig 7A). The process of multi-needle deployment and application of the C-plane are explained in detail in S1–S4 Videos.

**Fig 8:**
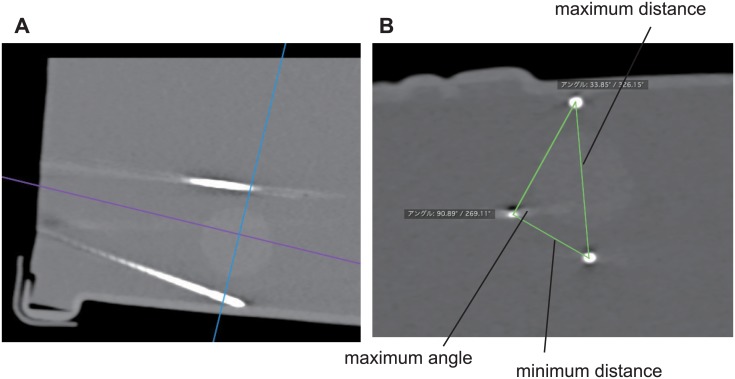
Definitions of angles and distance ratios. A) Computed tomography was performed immediately after the puncture procedure. B) The blue line represents the parasagittal plane. Each angle and distance between electrodes was measured.

Moreover, to assess the appropriate placement of the three electrodes in terms of the skill of the operator, puncture procedures both with and without the simulator were performed by two experts (M.H.: 15 years of experience in percutaneous procedures with 1,890 treatments, and Y.K.: 10 years of experience with 781 treatments), two non-experts (Y.I.: 5 years of experience with 116 treatments, and T.W.: 10 years of experience with 122 treatments), and two beginners (O.Y.: 2 years of experience with 13 treatments, and T.M.: one year of experience with 11 treatments), as in a previous study [17]. Punctures with the three electrodes both with and without the simulator were repeated 10 times by each operator (total, 120 times). Technical success was defined in terms of the maximum angle and distance ratio (Fig 8). Distance ratio was calculated by dividing maximum distance by minimum distance. The time required for placement of the three electrodes was measured.

To establish the target performance standard for insertion of 2 and 3 electrodes, 10 punctures were performed by the expert (M.I.) for each type of electrode, which included three monopolar electrodes (Cool-tip; Radionics, Burlington, MA) and three bipolar electrodes.

### Statistical analysis

Statistical analyses were performed using JMP version 8 software (SAS Institute Japan, Tokyo, Japan). To assess accuracy in the phantom study, correlations between data from each imaging modality were analyzed using Spearman correlation coefficients. Correlations between ablation volume and the imagined sphere were also analyzed by Spearman correlation coefficients. Differences in median values were assessed with the Steel-Dwass multiple comparison procedure. Values of *P*<0.05 were considered significant.

## Results

**Fig 9:**
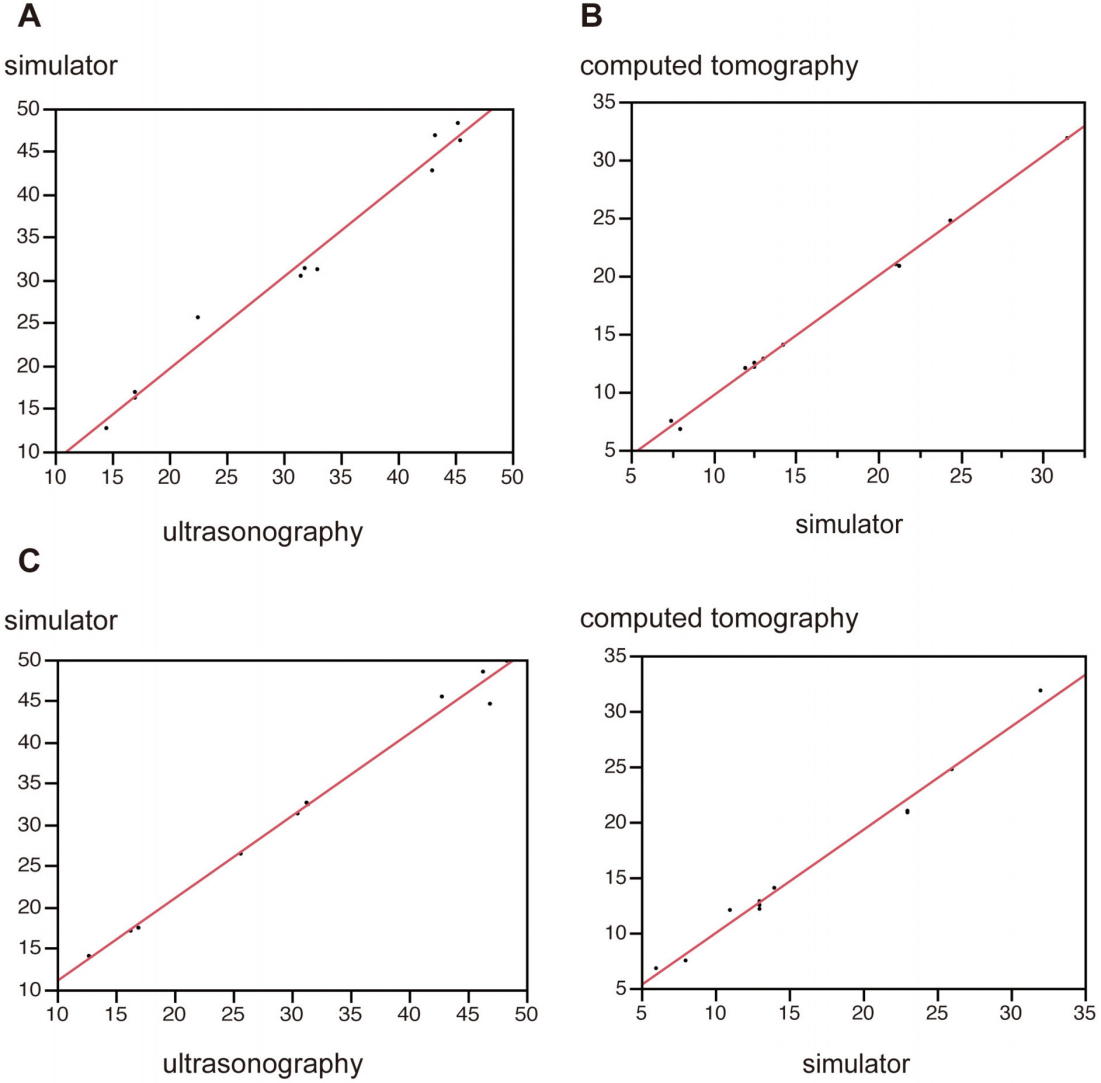
Analysis after insertion of 2 electrodes. **A, B)** Angles of intersection of the 2 electrodes were measured (**A:** US vs. simulator; **B:** simulator vs. CT). **C, D)** Distances between electrodes were also measured. Both angles and distances strongly correlated between images.

**Fig 10:**
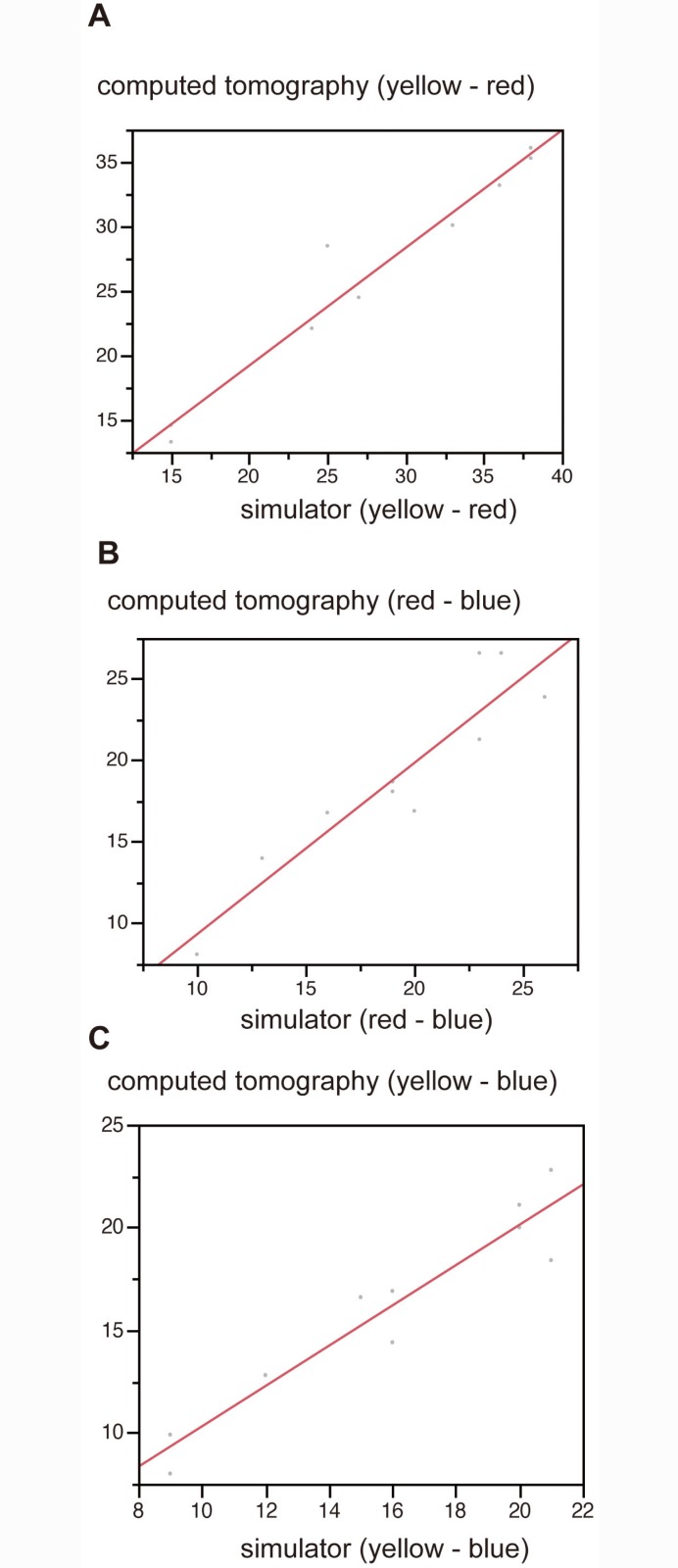
Analysis after insertion of the 3 electrodes. The distances between each electrode are shown (A: yellow—red, r = 0.98, p<0.0001; B: red—blue, r = 0.927, p<0.0001; C: yellow—blue, r = 0.953, p<0.0001). There was a good correlation between simulator and CT images.

In the analysis of angles between electrodes, interobserver reproducibility of values measured by the two examiners was high (US: r = 0.997, p<0.001; simulator: r = 0.996, p<0.001; CT: r = 0.995, p<0.001). Correlations between angles on each imaging modality were also strong (US vs. simulator: r = 0.991, p<0.001, simulator vs. CT: r = 0.991, p<0.001, US vs. CT: r = 0.999, p<0.001) (Fig 9A and 9B). Distances between electrodes were analyzed in a similar manner. In the analysis of distances, reproducibility was strong (US: r = 0.991, p<0.001; CT: r = 0.997, p<0.001). Correlations between distances in each imaging modality were also strong (US vs. simulator: r = 0.993, p<0.001; simulator vs. CT: r = 0.994, p<0.001; US vs. CT: r = 0.994, p<0.001) (Fig 9C and 9D). In cases of punctures with 3 electrodes, the distances between each electrode correlated strongly (yellow-labeled vs. red-labeled: r = 0.980, p<0.001; red-labeled vs. blue-labeled: r = 0.953, p<0.001; yellow-labeled vs. blue-labeled: r = 0.953, p<0.001) (Fig 10).

**Fig 11:**
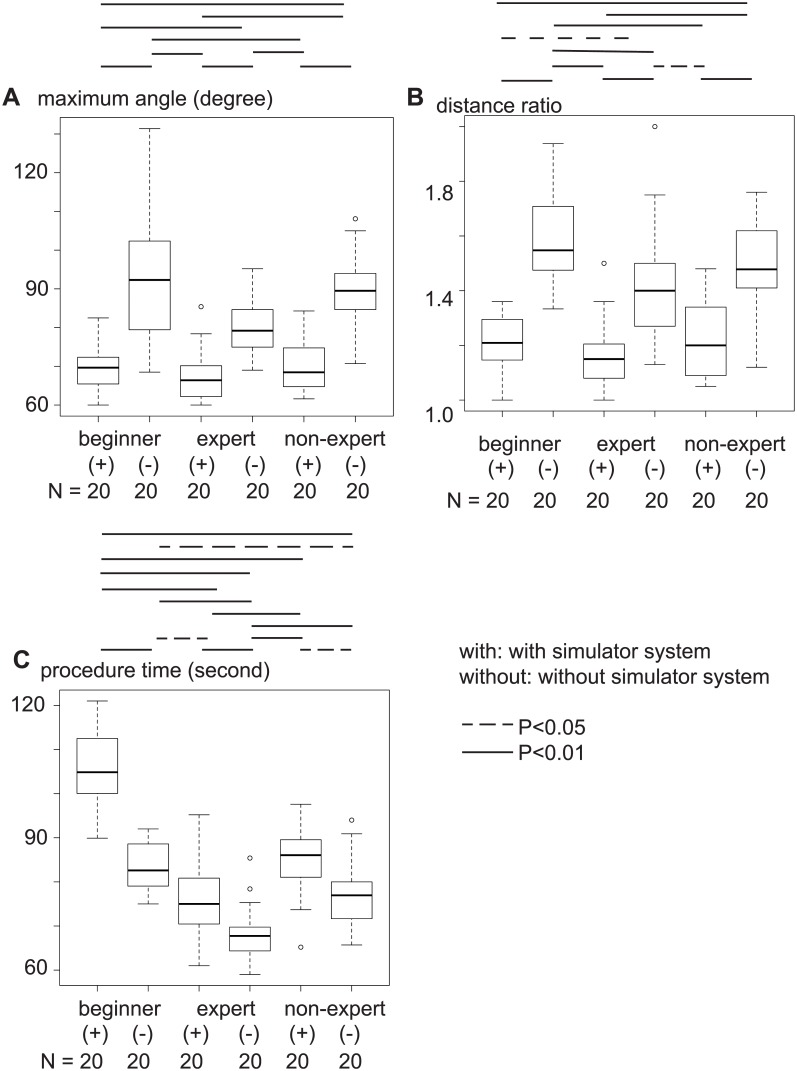
Maximum angle, distance ratio, and treatment time with expert or non-expert operators. **A)** With all the operators, maximum angles were significantly smaller when using the simulator system than without the simulator system. **B)** With all the operators, distance ratio was also significantly smaller when using the simulator system than without the simulator system. **C)** Treatment time required for punctures was significantly shorter with an expert user.

**Fig 12:**
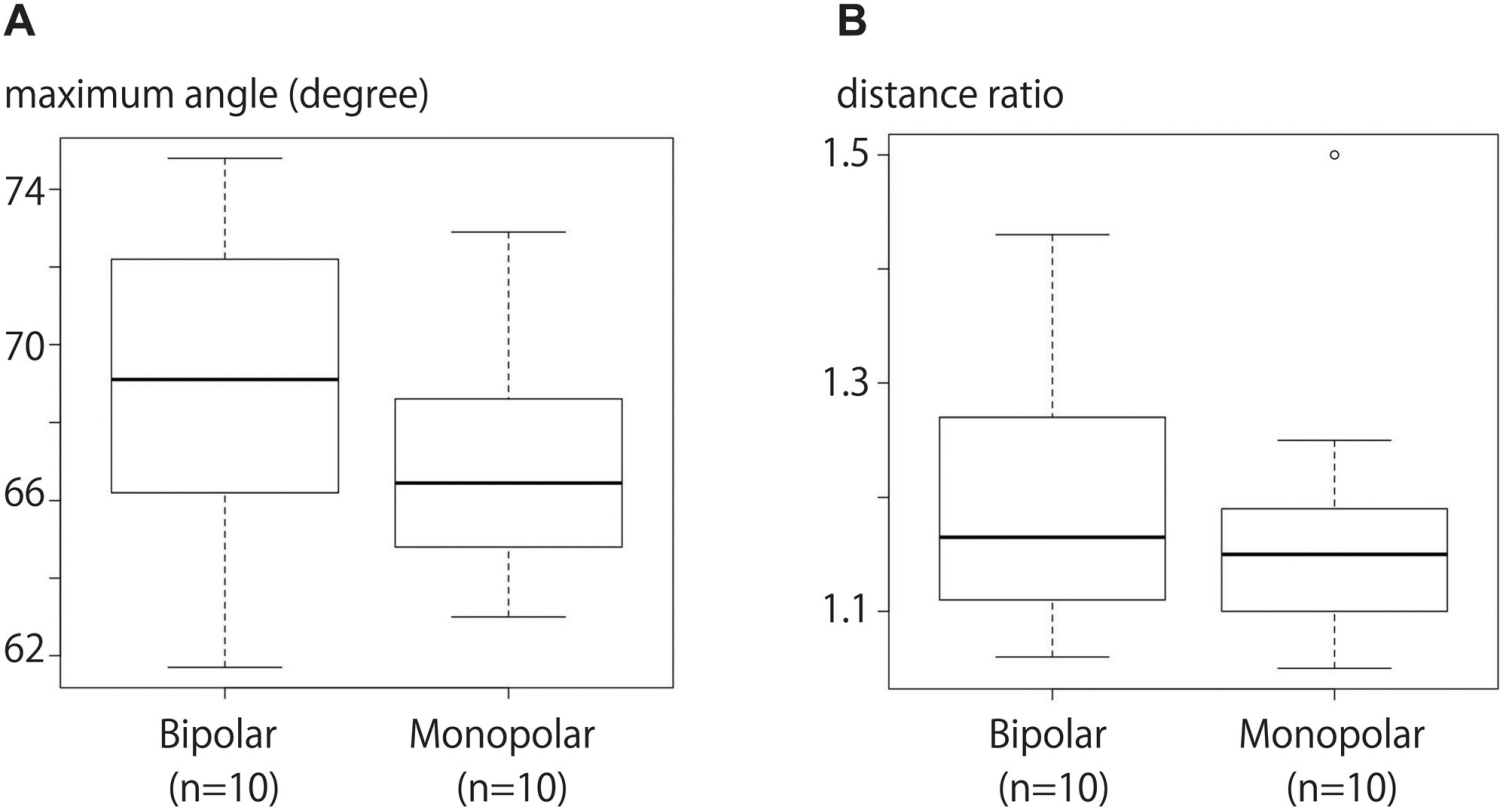
Maximum angle and distance ratios with the 2 types of electrodes. **A)** Maximum angles did not differ significantly between the 2 types of electrodes. **B)** Distance ratio also did not differ significantly.

Maximum angle and distance ratio were measured to evaluate the placement of electrodes as guided by the simulator system. Both angle (expert with simulator vs. without simulator, p<0.01; non-expert, <0.01; beginner, <0.01) and distance ratio (expert with simulator vs. without simulator, p = 0.01; non-expert, <0.01; beginner, <0.01) were significantly smaller in the procedures involving use of the simulator system by all types of operators (Fig 11). With the simulator system, whether the operator was an expert or beginner, neither angle (expert vs. non-expert, p = 0.74; non-expert vs. beginner, P = 1.00; beginner vs. expert, P = 0.83) nor distance ratio (expert vs. non-expert, p = 0.74; non-expert vs. beginner, P = 1.00; beginner vs. expert, P = 0.58) were significantly different. Using this system, procedure time when performed by an expert was shorter than that with a non-expert and beginner (expert vs. non-expert, p = 0.03; non-expert vs. beginner, P<0.01; beginner vs. expert, P<0.01). Angle and distance ratios did not differ significantly between monopolar and bipolar electrodes (angle, p = 0.289; distance ratio, p = 0.544; Fig 12).

## Discussion

We developed a simulator system for use during multipuncture ablation. In the phantom study, reproducibility between virtual imaging with the simulator system and real imaging by CT was very good.

A simulator system to facilitate multipuncture procedures is warranted especially for ultrasound-guided procedures, though it might be not useful for CT-guided procedures. We developed the simulator system based on the concept of the virtual puncture line, to understand the placement of each electrode [15]. Two new concepts are incorporated into this system. First, the radiofrequency electrode can be visualized in 3D images. Understanding the positions and directions of each electrode can be very difficult, because the electrodes are not inserted in parallel at regular intervals. Using the simulator, the positions and directions of insertion of two or three electrodes can be more easily understood. Second, the concept of the C-plane was presented to optimize the multipuncture procedure. The C-plane is defined as the sagittal plane perpendicular to the depicted plane. Particularly in the case of multipuncture ablation with three radiofrequency electrodes, the C-plane appears to be very useful. It is important that the electrodes pass through appropriate site at the largest cross-sectional area of the tumor. Moreover, the three electrodes should be inserted so as to form the apices of an equilateral triangle. From this perspective, visualization of the site of insertion of each electrode in the C-plane is extremely helpful. This study evaluated the accuracy of the simulator. MPR images constructed from CT were used as the reference standard, because angles and distances could not be measured *in vivo*. In the first analysis, correlations between distances and angles in each imaging modality were strong for the two experts. In cases of puncture with both 2 and 3 electrodes, reproducibility was very strong using the simulator system. Subsequently, accuracy for the placement of electrodes was assessed by determining whether reproducibility differed according to operator experience, type of electrode, or use of the simulator system. In this study, maximum angle and distance ratio were used to estimate appropriate placement of the electrodes as the vertices of an equilateral triangle. With this positioning, the maximum angle would be close to 60°. Furthermore, the distance ratio was close to 1.0. The merits of this system were clearly shown with all users in the first study, whether expert or not. Interestingly, beginners achieved adequately accurate punctures with this system, although, not surprisingly, they took a longer time to achieve adequate puncture. Thus, using the phantom, if a suitably long time is available for performance of the procedure, beginners may be able to perform treatments with this system comparable to those provided by experts. The system is, thus, beneficial for all operators.

This study has several limitations. First, the efficacy of no-touch ablation has yet to be established in a prospective controlled trial. Seror et al. reported the advantage of no-touch ablation in a retrospective study [15]. Hence, a randomized controlled trial or cohort study should be performed to evaluate this. However, this simulator is expected to be useful for large tumors. Second, although this system was tested by two experts, two non-experts, and two beginners in this study, all of them were previously trained in the use of the phantom. Participants, therefore, became accustomed to controlling this system. Further, more than ten punctures were performed by each user. This suggests that this system cannot be considered ready to use without appropriate training. Third, this study was not applied in vivo. Clarification of RFA needle puncture in vivo is clearly needed. Phantoms lack the confounding influence of respiration and the presence of blood flow and bile ducts. The present study only proved that this system offers good reproducibility as an imaging system. More detailed analysis of the utility of RFA guidance using this system is required under clinical situations. Moreover, in clinical situations, the beginner will presumably not be able to operate this system in the same manner as in the phantom study. Fourth, this simulator system is only applicable to US-guided RFA, not CT-guided RFA.

## Conclusions

The accuracy of our simulator system was suggested from this phantom study. In multipuncture ablation, this simulator system appears to be very useful for understanding the placement and direction of RFA electrodes. Since RFA with multiple electrodes needs a high level of skill, this simulator system could make the multipuncture procedure more effective and more comfortable for doctors treating HCC.

## Supporting Information

S1 Video C-plane and 3D image. In 3D images, the blue plane is defined as the virtual ultrasound image. The light-green line in the blue plane is the puncture line. The C-plane is the sagittal plane of the blue plane and the x mark is defined as the intersection between the light green line and the sagittal plane.
(MP4)

S2 Video Puncture by the first electrode. The inserted electrode is shown as a yellow line, using the same method applied in Fig 2.
(MP4)

S3 Video Puncture with the second electrode. After confirming the position of the first electrode, the second electrode was inserted. This electrode was marked as a yellow line, using the same method as in Fig 5.
(MP4)

S4 Video Puncture with the third electrode. After confirmation of the position of the first and second electrodes, a third electrode was inserted.
(MP4)

## References

[1] Woo S, Lee JM, Yoon JH, Joo I, Kim SH, Lee JY, et al Small- and medium-sized hepatocellular carcinomas: monopolar radiofrequency ablation with a multiple-electrode switching system-mid-term results. Radiology 2013;268: 589–600. 10.1148/radiol.13121736 23513241

[2] Cady B, Jenkins RL, Steele GD Jr, Lewis WD, Stone MD, Lee JY, et al Surgical margin in hepatic resection for colorectal metastasis: a critical and improvable determinant of outcome. Ann Surg. 1998;227: 566–571. 9563547 10.1097/00000658-199804000-00019 PMC1191314

[3] Elias D, Cavalcanti A, Sabourin JC, Pignon JP, Ducreux M, Lasser P. Resection of liver metastases from colorectal cancer: the real impact of the surgical margin. Eur J Surg Oncol. 1998;24: 174–179. 9630855 10.1016/s0748-7983(98)92878-5

[4] Nakazawa T, Kokubu S, Shibuya A, Ono K, Watanabe M, Hidaka H, et al Radiofrequency ablation of hepatocellular carcinoma: correlation between local tumor progression after ablation and ablative margin. AJR Am J Roentgenol. 2007;188: 480–488. 17242258 10.2214/AJR.05.2079

[5] Dodd GD 3rd, Frank MS, Aribandi M, Chopra S, Chintapalli KN. Radiofrequency thermal ablation: computer analysis of the size of the thermal injury created by over- lapping ablations. AJR Am J Roentgenol. 2001;177: 777–782. 11566672 10.2214/ajr.177.4.1770777

[6] Lencioni R, Cioni D, Bartolozzi C. Percutaneous radiofrequency thermal ablation of liver malignancies: techniques, indications, imaging findings, and clinical results. Abdom Imaging 2001;26: 345–360. 11441546 10.1007/s002610000194

[7] Seror O, N'Kontchou G, Ibraheem M, Ajavon Y, Barrucand C, Ganne N, et al Large (≥5.0-cm) HCCs: multipolar RF ablation with three internally cooled bipolar electrodes—initial experience in 26 patients. Radiology 2008;248: 288–296. 10.1148/radiol.2481071101 18483229

[8] Osaki Y, Ikeda K, Izumi N, Yamashita S, Kumada H, Hatta S, et al Clinical effectiveness of bipolar radiofrequency ablation for small liver cancers. J Gastroenterol. 2013;48: 874–883. 10.1007/s00535-012-0685-x 23053425

[9] Frericks BB, Ritz JP, Roggan A, Wolf KJ, Albrecht T. Multipolar radiofrequency ablation of hepatic tumors: initial experience. Radiology 2005;237: 1056–1062. 16237132 10.1148/radiol.2373041104

[10] Clasen S, Schmidt D, Boss A, Dietz K, Kröber SM, Claussen CD, et al Multipolar radiofrequency ablation with internally cooled electrodes: experimental study in ex vivo bovine liver with mathematic modeling. Radiology 2006;238: 881–890. 16424244 10.1148/radiol.2382050571

[11] Seror O, N'Kontchou G, Van Nhieu JT, Rabahi Y, Nahon P, Laurent A, et al Histopathologic comparison of monopolar versus no-touch multipolar radiofrequency ablation to treat hepatocellular carcinoma within Milan criteria. J Vasc Interv Radiol. 2014;25: 599–607. 10.1016/j.jvir.2013.11.025 24529547

[12] Yu SC, Lo DY, Ip CB, Liew CT, Leung TW, Lau WY. Does percutaneous liver biopsy of hepatocellular carcinoma cause hematogenous dissemination? An in vivo study with quantitative assay of circulating tumor DNA using methylation-specific real-time polymerase chain reaction. AJR Am J Roentgenol. 2004;183: 383–385. 15269029 10.2214/ajr.183.2.1830383

[13] Takamori R, Wong LL, Dang C, Wong L. Needle-tract implantation from hepatocellular cancer: is needle biopsy of the liver always necessary? Liver Transpl. 2000;6: 73–75. 10648580 10.1002/lt.500060103

[14] Chapoutot C, Perney P, Fabre D, Taourel P, Bruel JM, Larrey D, et al Needle-tract seeding after ultrasound-guided puncture of hepatocellular cancer: a study of 150 patients. Gastroenterol Clin Biol. 1999;23: 552–556. 10429862

[15] Hirooka M, Kisaka Y, Uesugi K, Koizumi Y, Abe M, Hiasa Y, et al Virtual puncture line in radiofrequency ablation for hepatocellular carcinoma of the caudate lobe. AJR Am J Roentgenol. 2009;193: W149–151. 10.2214/AJR.08.1817 19620418

[16] Matsuda M, Tsuda T, Yoshioka S, Murata S, Tanaka H, Hirooka M, et al Incidence for progression of hypervascular HCC in hypovascular hepatic nodules showing hyperintensity on gadoxetic acid-enhanced hepatobiliary phase in patients with chronic liver diseases. Jpn J Radiol. 2014;32: 405–413. 10.1007/s11604-014-0323-z 24854900

[17] Kang TW, Lee MW, Choi SH, Rhim H, Lim S, Song KD, et al A novel electrode with electromagnetic tip tracking in ultrasonography-guided radiofrequency ablation: a phantom, ex vivo, and in vivo experimental study. Invest. Radiol. 2015;50: 81–87. 10.1097/RLI.0000000000000103 25360604

